# Exosome-transmitted circular RNA circ-LMO7 facilitates the progression of osteosarcoma by regulating miR-21-5p/ARHGAP24 axis

**DOI:** 10.1080/15384047.2024.2343450

**Published:** 2024-05-14

**Authors:** Anyu Luo, Hanlin Liu, Chen Huang, Sheng Wei

**Affiliations:** Department of Orthopedics, Hanyang Hospital Affiliated to Wuhan University of Science and Technology, Wuhan, Hubei, China

**Keywords:** Osteosarcoma, circ-LMO7, miR-21-5p, ARHGAP24, exosome

## Abstract

The potential function and mechanism of circRNAs in regulating malignant performances of Osteosarcoma (OS) cells have not been well investigated. The expression level of CircLMO7, miR-21-5p and ARHGAP24 were detected by RT-qPCR. The relationship between miR-21-5p and circ-LMO7, as well as between miR-21-5p and ARHGAP24, was predicted and examined through bioinformatics analysis and luciferase reporter gene experiments. Moreover, OS cell growth, invasion, migration, and apoptosis were detected using the cell counting kit-8 (CCK-8), transwell and flow cytometry assays, respectively. ARHGAP24 protein level was measured using western blotting. In present study, we choose to investigate the role and mechanism of circ-LOM7 on OS cell proliferation, migration and invasion. circ-LOM7 was found to be down-regulated in OS tissues and cell lines. Enforced expression of circ-LOM7 suppressed the growth, invasion, and migration of OS cells. In contrast, decreasing circ-LMO7 expression had opposite effects. Furthermore, miR-21-5p was predicted to be sponged by circ-LMO7, and had an opposite role of circ-LMO7 in OS. Moreover, ARHGAP24 served as miR-21-5pʹs downstream target. Mechanistically, circ-LMO7 was packed in exosomes and acted as a cancer-suppresser on OS by sponging miR-21-5p and upregulating the expression of ARHGAP24. The exosomal circ-LMO7 expression was significantly decreased in OS cell exosomes, and co-culture experiments showed that exosomal circ-LMO7 suppressed the proliferation ability of OS cells. Circ-LMO7 exerts as a tumor suppressor in OS, and the circ-LMO7/miR-21-5P/ARHGAP24 axis is involved in OS progression.

## Introduction

Osteosarcoma (OS) is a common bone cancer which was mostly diagnosed among young patients. Most obvious and important features of OS are prone to lung metastasis, progresses rapidly, and morbidity and mortality are high.^1^ Due to surgery, chemotherapy, immunotherapy and gene therapy, clinical prognosis of osteosarcoma patients has been well improved.^2,3^ But OS patients still have a poor survival rate at 5 years, which seriously threatens people’s health.^4,5^ Given this, it is urgent to identify new and efficient diagnostic/prognostic biomarkers and therapeutic targets for OS patients to develop new treatment strategies to further improve OS survival.^6,7^

CircRNA is described as one type of non-coding RNAs which could not encode protein and used to be considered as the noise of genomic transcription without any biological function for a long time.^8^ However, dysregulated circRNAs have been observed in multiple tumors, such as colon cancer,^9^ pancreatic cancer,^10^ cancer, and breast cancer.^11^ The potential function and mechanism of circRNA in regulating malignant performances of tumor cells have been well investigated. Recent evidences have supposed that circRNA not only directly regulates gene expression, but also acts as ceRNA (competing endogenous RNA) to interacts with miRNA. For example, wang’s study revealed that circACTN4 mediated by USF2 might interact with FUBP1 to promote progression of breast cancer via enhancing the expression of MYC.^12^ CircDOCK1 promotes tumorigenesis and cisplatin resistance in osteosarcoma via the miR-339-3p/IGF1R axis.^13^ CircAGO2, up-regulated in various cancer and associated with poor prognosis of patients, drives cancer progression through facilitating HuR-repressed functions of AGO2-miRNA complexes.^14^ CircPIP5K1A was significantly upregulated and promotes tumorigenesis and aggressiveness of colon cancer.^15^ Despite the achieved advances, circRNA in osteosarcoma is rarely explored and need further exploration.

Previous study has revealed that hsa_circ_0004872 was dramatically downregulated in gastric cancer tissues and increased hsa_circ_0004872 expression inhibited the proliferation, invasion and migration of GC cells via miR-224/Smad4/ADAR1 pathway.^16^ In this study, we examined the expression pattern of hsa_circ_0004872 and elucidated its biological function in the development of OS.

## Material and methods

### Bioinformatics analysis

The OS GEO databases (GSE96964) was searched to obtain circRNAs. The above circRNA expression profile cohorts were normalized based on the Robust Multi-Array Average (RMA) and Linear Models for Microarray (LIMMA) algorithm. The DEMs between OS cases and the normal cases was explored based on Limma package. *p* < .05 and log_2_FC > 1.3 were considered to be statistically significant. After that, the volcano map was performed via ggplot2 package. The identified miRNAs was applied to predict their target genes based on miRWalk2.0.^17^ The identified targeted genes were used for PCR and western blot confirmation.

### Cell culture and transfection

The OS cell lines Saos-2, MG63, MNNG/HOS and U-2OS and Human osteoblast hFOB 1.19 were purchased from the American Type Culture Collection (Manassas, VA). All the cells were cultured in a humidified atmosphere 5% carbon dioxide at 37°C incubator containing RPMI 1640 supplemented with 10% FBS, penicillin (100 U) as well as streptomycin (100 µg/mL).

The miR-21-5p mimic and the mimics negative control (miR-NC) were designed and synthesized by Sangon Biotech Ltd. The pcDNA (Vector), pcDNA-circ-LOM7 and pcDNA-ARHGAP24 plasmids were designed to be applied. The mimic and plasmids were used for the transfection of MNNG/HOS and MG63 applying Lipofectamine 3000 (Invitrogen) for 48 h on the basis of the instructions of the manufacturer.

### RNA extraction and qRT-PCR

Trizol reagent (Carlsbad, USA) was used to extracted Cells total RNA. Then, RNA was reversely transcribed into by using RT reagent Kit (Takara). Next, qRT-PCR was conducted employing the SYBR Green PCR Master Mix (Transgen, Beijing, China) in accordance to the instructions of the manufacturer. The specific primer sequence was listed in Table 1. The circ-LOM7, miR-21-5p or ARHGAP24 mRNA level was assessed with the 2-ΔΔCt approach that were normalized via U6 or GAPDH, respectively.

**Table 1.** Primers used for RT-qPCR in the present study.

Gene	Forward primer	Reverse primer
CircLMO7	ACAGATTGGATTGAAAGAAGCCC	CACACAGCAGAACACCATTTTC
CircPAPPA	ATCGATGCTGCCATGTTGAC	GTGTGGGTTGACAGCTGAAT
hsa_circ_0027493	ACCTCATCTAGAAGGAGAGCA	TCCTTTTGATCACTCCCACCT
hsa_circ_0007747	CCCCAGAGCTGCATCCTTAT	ATCTGCTGCAACCTGTGATG
hsa_circ_0002052	ATCGATGCTGCCATGTTGAC	CTACTCCTGCCAACTCCTCC
hsa_circ_0008792	AAGGTGAACAATGCGACGAC	TGGGTTCAAGACAATGCCAC
hsa_circ_0088214	GCATGTCATCTTTGCCTGGA	TGGGTTCAAGACAATGCCAC
hsa_circ_0087283	CAACAAGTGGTGCAGGCTG	TGGGACAGGAAAGGAATGACT
hsa_circ_0007610	TGGATTGAAAGAAGCCCAGC	AATGACGCCAGGTTTAAGCT
hsa_circ_0088209	GATGAGCACCTGGAGATCGA	GCTCGGTCTGTCTTCAAGGA
LMO7	GAAAATGGTGTTCTGCTGT	CTGTAGATCTCCAGGATGG
PAPPA	CCTGGGTCCTCAGAATGTC	AGGTGCTCATCCAGCGTGT
hsa-miR-21-5p	CGGCGCAACACCAGTCGATG	AGTGCAGGGTCCGAGGTATT
hsa-miR-590-5p	GAGCTTATTCATAAAAGTG	TGACCCCAGGTAACTCTGAGTGTGT
hsa-miR-378 g	TGTGGGCATCAATGGATTTGG	ACACCATGTATTCCGGGTCAAT
GAPDH	TGTGGGCATCAATGGATTTGG	ACACCATGTATTCCGGGTCAAT
ARHGAP24	GAACCGTCTGGCTCCGATG	TGGCAGTCGAAAGAGACCCT
EPHA4	TTCGCCCTATTTTCGTGTCTC	TGGTAGGTTCGGATTGGTGTAT
PITX2	CGGCAGCGGACTCACTTTA	GTTGGTCCACACAGCGATTT
THRB	TGGGACAAACCGAAGCACTG	TGGCTCTTCCTATGTAGGCAG
MAP3K1	CCTTCTACGACGCAGATGTTG	GCATCGGTGTCATGGTACAAGA
ADGRG2	CTGTGGCCTATGGCGTAGC	GCTCGTCGAAGAGACCCTG

### Western blot

Cells were transfected and collected from all groups, then lysed using RIPA buffer (ShineGene Molecular Biotech, Inc., Shanghai, China). Total proteins were extracted based on the manufacturer’s instructions. Subsequently, proteins were separated with 10% SDS – PAGE and then transferred to polyvinylidene fluoride (PVDF) membranes (Invitrogen). Then, the membranes were blocked using 5% nonfat milk and incubated with primary antibodies (GAPDH, 1:5000, ab8245; ARHGAP24, 1:500, ab203874) at 4°C overnight. Following that, the bands were detected chemiluminescence reagent (Millipore, USA) in ChemiDoc System (Bio-Rad, USA).

### Luciferase reporter assay

The synthetic wild-type and mutated binding sites of miR-21-5p in circ-LOM7 or ARHGAP24 sequences were inserted into the pmirGLO vector (Promega, Madison, WI, USA). The reporter plasmids circ-LOM7-WT/Mut and ARHGAP24–3′-UTR-WT/Mut were constructed and transfected into MNNG/HOS and MG-63 cells. The Dual-Glo® Luciferase Assay System (Promega) was adopted to access the luciferase intensity.

### Transwell assay

The invasive and migratory abilities of MG63, MNNG/HOS cells were tested through transwell assay. Transfected PC cells were added into the upper chamber insert with coated Matrigel (1 μg/ml) (Sigma-Aldrich, USA). The lower compartment insert culture medium containing 10% FBS. After incubation for 24 h, the cells remaining in the upper compartment were cleared away, meanwhile cells diffusing into the membrane were fixed using methyl alcohol and stained applying 0.1% crystal violet. Eventually, the counting for the stained cells was conducted with a bright-field microscope.

### Colony formation assay

A 3-(4,5-dimethylthiazol-2-yl)-2,5-diphenyltetrazolium bromide (MTT) assay was implemented to detect the proliferation of target cells according to the manufacturer’s recommendations. Cells were seeded on a 96-well plate at a density of 5 × 10^3^ cells/well which were cultured for 1 to 5 days at 37°C. At indicated time, the cells was incubated with 20 μl MTT for 4 h at 37°C. Then, the culture medium was wiped out and 150 μl DMSO (Sigma) was added to every well for 30 min at room temperature. Eventually, the optical density (OD) was evaluated at a 450 nm wavelength.

### CCK-8

After transfection, the MNNG/HOS and MG-63 cells were cultured in 96-well plates. Fresh complete culture medium (100 μL) was replaced at an interval of 24 h (90 μL fresh complete culture and 10 μL CCK-8 solution into all wells), followed by incubation in the incubator for another 1 h. A microplate analyzer was used to determine absorbance at 450 nm (A450).

### Flow cytometry

Apoptosis was detected using an Annexin V-FITC/PI apoptosis assay kit. At 48 h after transfection, MNNG/HOS and MG-63 cells were digested with 0.25% trypsin and re-suspended twice with binding buffer. Thereafter, every tube was added with Annexin V-FITC (5 µL) and propidium iodide (PI, 10 μL) for 10 min, followed by incubation at ambient temperature. Finally, cell apoptosis was detected by flow cytometry after 1 h.

### Exosomes isolation

Exosomes were isolated from the supernatant of OS cells and hFOB 1.19. Cells. Isolation of exosomes was performed in accordance with a previous study.^18^ Nanoparticle tracking analysis (NTA) 2.3 software (Shanghai XP Biomed Ltd., Shanghai, China) was used for later analysis the isolated exosomes.

### Statistical analysis

Statistical analyzes were performed applying SPSS. All results were registered as mean ± standard deviation. The significance of differences between two groups was evaluated via Student’s t-test, while those among multiple groups were evaluated by One-way ANOVA. Prognostic significances were assessed by Kaplan-Meier. All experiments were performed 3 times. *p* value < .05 was considered to be statistically significant.

## Result

### Circ-LMO7 was highly expressed in osteosarcoma cell lines

**Figure 1.**
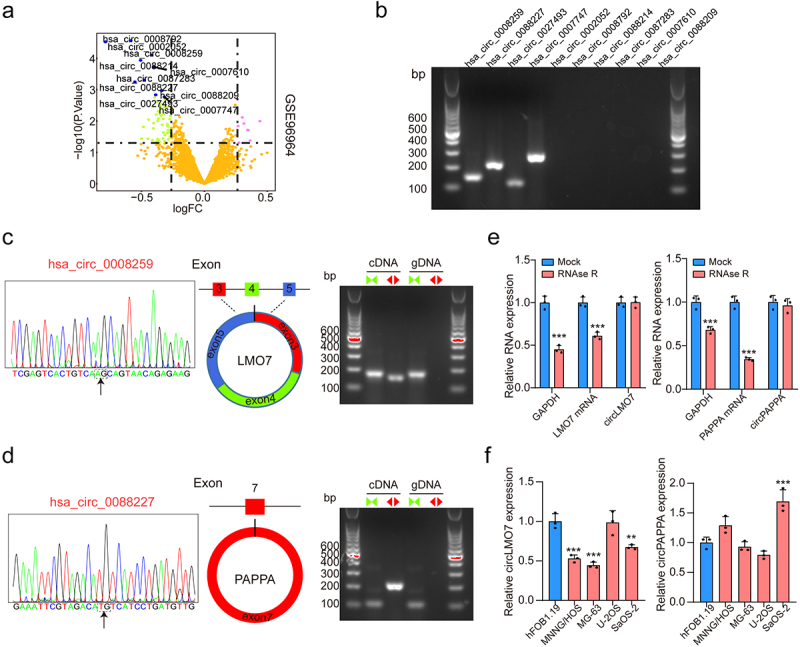
Screening and characterization of circ-LMO7.

To find the potential circRNAs in OS development, circRNA dataset containing 10 CRC patients (GSE96964) from GEO database was selected. As listed in Figure 1(a), top 10 circRNAs were screened out and identified. Among them, four circRNAs were validated by PCR amplification using divergent primers from cDNA in OS cell lines (Figure 1(a)). We then conducted sanger sequencing using the PCR products of these four circRNAs, and the results revealed that hsa_circ_0088259 (circ-LOM7) and hsa_circ_0088227 (circ-PAPPA) were consistent with the sequence information in the circBase database (Figure 1(c,d)). In addition, we found that the linear form of GAPDH was dramatically reduced after RNase R administration, while circ-LOM7 and circ-PAPPA resisted RNase R digestion, indicating circ-LOM7 and circ-PAPPA are bona fide circRNAs in OS cell (Figure 1(e)). Next, the expressions of circ-LOM7 and circ-PAPPA were measured in different OS cell lines (MNNG/HOS, MG-63, U-2OS and SaOS-2 cells). As shown in Figure 1(f), the expression level of circ-LOM7 was decreased in most of OS cell lines, while circ-PAPPA did not have a different expression. Based on above results, we supposed that circ-LOM7 plays significant role in the tumorigenesis of OS and was selected for further analysis.

(a) Top 10 dysregulated CircRNAs in GSE96964. (b) DNA electrophoresis was used to detect the size of circRNAs. (c) Circ_0008259 was derived from LMO7 mRNA and the circular structure of circ_0008259 was verified by PCR assays. (d) Circ_0088227 was derived from PAPPA mRNA and the circular structure of circ_0088227 was verified by PCR assays. (e) The expressions of circ-LMO7, LMO7 mRNA, circ-PAPPA, PAPPA mRNA and GAPDH were identified by qRT-PCR after treatment with RNase R. (f) Circ-LMO7 and circ-PAPPA expression in OS cell lines (MNNG/HOS, MG-63, U-2OS and SaOS-2) and the hFOB1.19 cells was examined by qRT-PCR. *p < .05, **p < .01, ***p < .001

### Circ-LMO7 inhibited the proliferation and migration of osteosarcoma cells

**Figure 2.**
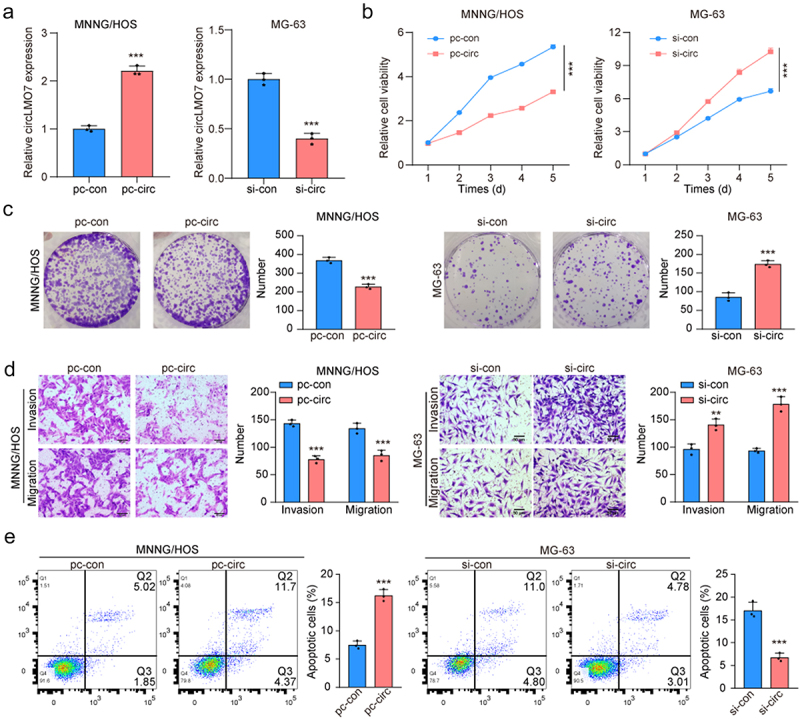
The inhibiting effects of circ-LMO7 in OS development.

Since circ-LOM7 expression was decreased in OS cell lines, pc-circ-LOM7 and siRNA for circ-LOM7 was transfected into MNNG/HOS and MG-63 cells to enhance and downregulate the circ-LOM7 expression (Figure 2(a)). As shown in Figure 2(b), the viability of MNNG/HOS and MG-63 cells was decreased and increased with the pc-circ-LOM7 and siRNAs transfection, respectively, indicating circ-LOM7 over-expression inhibited the proliferation of OS cells. circ-LOM7 over-expression also reduced the number of clones of MNNG/HOS and MG-63 cells (Figure 2(c)). In addition, the migratory and invasive abilities of MNNG/HOS and MG-63 cells were damaged after upregulation of circ-LOM7 expression (Figure 2(d)). Moreover, we found circ-LOM7 overexpression promoted the apoptotic rate of MNNG/HOS and MG-63 cells (Figure 2e).

(a) The transfection efficiency of circ-LMO7 plasmid and siRNAs was verified by qRT-PCR. (b, c) Cell proliferation was determined by colony formation assays and CCK-8 in transfected MNNG/HOS and MG-63 cells (d) The representative results of the Transwell assay show changes in the migration and invasion capacities of in transfected MNNG/HOS and MG-63 cells (scale bar, 200 μm). (e) Transfected MNNG/HOS cells and MG-63 cells apoptosis were detected through flow cytometry. *p < .05, **p < .01, ***p < .001

### Circ-LMO7 interacts with miR-21-5p

**Figure 3.**
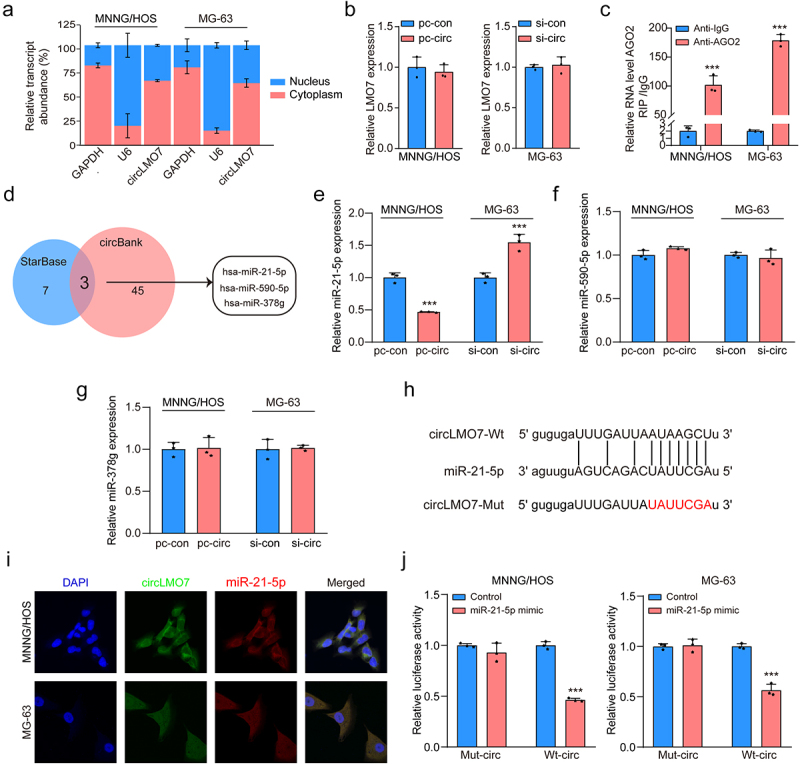
Circ-LMO7 directly targets on miR-21-5p in OS cells.

Previous studies have shown that the function of circRNA was related to the subcellular localization. We therefore investigated the cellular distribution of circ-LOM7. RT-qPCR results in MNNG/HOS and MG-63 cells revealed that circ-LOM7 predominantly localizes to the cytoplasm (Figure 3(a)).

(a, b) Subcellular localization of circ-TRIM35 in MNNG/HOS and MG-63 cells, as determined using PCR. (b) qRT – PCR analysis of LMO7 expression in transfected MNNG/HOS and MG-63 cells (c) The enrichment of circ-LMO7 in the immunoprecipitation products was detected by qPCR. (d) The miRNAs that may bind with circ-LMO7 were predicted through StarBase database and CircBank database. (e, f, & g) MiR-21-5p, miR-590-5p and miR-378 g expression in transfected MNNG/HOS and MG-63 cells was examined by qRT-PCR. (h) MiR‐21-5p binding sequences in circ-LMO7 are displayed, and reporter gene plasmids were constructed. (i) Dual RNA-FISH and immunofluorescence staining assays indicating the co-localization of circ-LMO7 (red) and miR-21-5p (green) in MNNG/HOS and MG-63 cells, with nuclei staining using DAPI (blue). (j) The regulatory relationships were confirmed by luciferase reporter assay. *p < .05, **p < .01, ***p < .001

Enforcing or depleting circ-LMO7 expression did not influence the expression level of its maternal gene LMO7 (Figure 3(c)), indicating that circ-LMO7 didn’t work in a cis-regulation manner. RNA immunoprecipitation (RIP) showed circ-LOM7 was enriched in MNNG/HOS and MG-63 cells treated with AGO2 antibody, suggesting that circ-LMO7 could directly target miRNAs in an AGO2 manner (Figure 3(c)). Thus, StarBase and Circbank databases were searched, and found that miR-21-5p, miR-590-5p and miR-378 might be downstream targets of circ-LOM7 (Figure 3(d)). The results of RT-qPCR showed that only the expression of miR-21-5p was remarkably decreased in both MNNG/HOS and MG-63 cells after circ-LOM7 overexpression (Figure 3(e–g)). The binding sites between circ-LOM7 and miR-21-5p were predicted by bioinformatics software (starBase) (Figure 3(h)). The outcome of dual RNA-FISH and immunofluorescence assays verified the colocalization of circ-LOM7 and miR-21-5p in MNNG/HOS and MG-63 cells (Figure 3(i)). The luciferase assay results showed that the relative activity of the WT reporter containing circ-LOM7 sequence but not the mutant reporter was obviously suppressed in cells co-transfected with the miR-21-5p mimic (Figure 3(j)), indicating miR-21-5p could directly bind to the binding sites of circ-LOM7.

### Circ-LMO7 inhibited the proliferation and migration of osteosarcoma cells via sponging miR-21-5p

**Figure 4.**
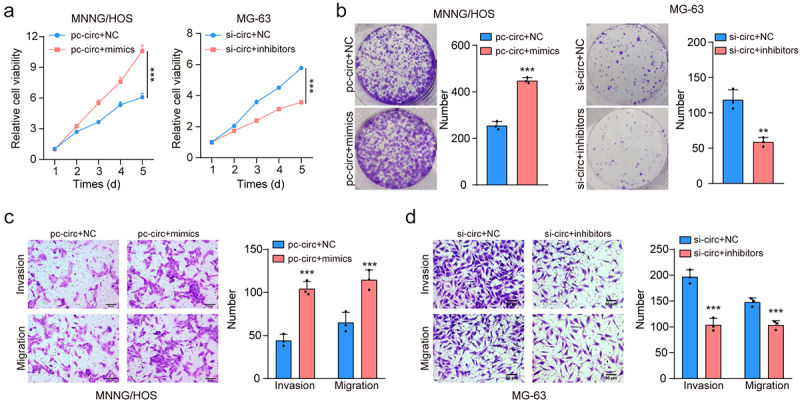
miR-21-5p reverse the inhibiting effects of circ-LMO7 in OS cells.

To explore the role of the circ-LOM7/miR-21-5p axis in OS, we transfected circ-LOM7-overexpressed MNNG/HOS cells with miR-21-5p mimic, whereas circ-LOM7-silencing MG-63 cells were transfected with the miR-21-5p inhibitor. The CCK-8, colony assays, transwell assays revealed that miR-21-5p overexpression could reverse the impacts of circ-LOM7 upregulation on MNNG/HOS cell growth, invasion, and migration. And on MG-63 cells, miR-21-5p downregulation partially reversed the effects induced by circ-LOM7 depletion (Figure 4(a–d)). Based on the above findings, the circ-LOM7/miR-21-5p axis is involved in OS progression.

(a, b) Cell proliferation was determined by CCK-8 and colony formation assays in transfected MNNG/HOS and MG-63 cells (c, d) The representative results of the Transwell assay show changes in the migration and invasion capacities of in transfected MNNG/HOS and MG-63 cells (scale bar, 200 μm). *p < .05, **p < .01

### Circ-LMO7 interacted with miR-21-5p and regulated ARHGAP24 expression

**Figure 5.**
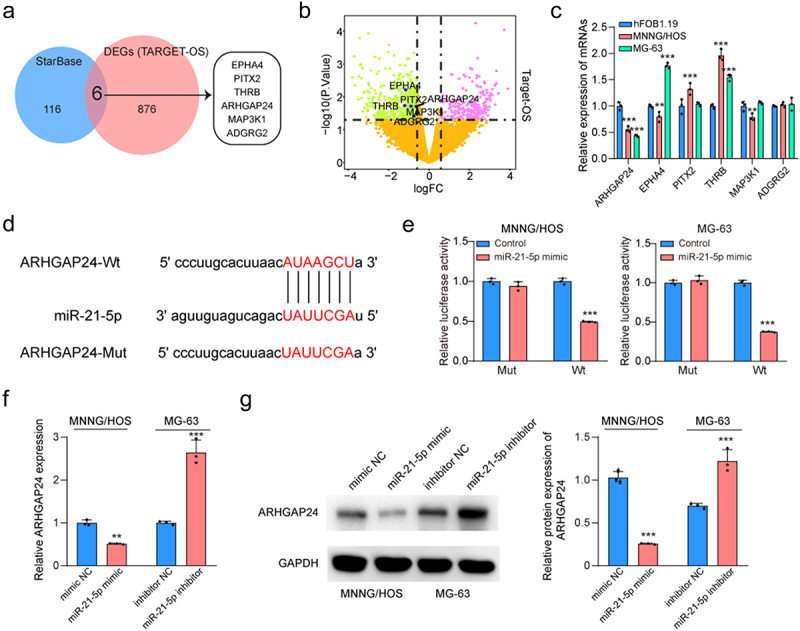
ARHGAP24 is a direct target of miR-21-5p.

Next, we explored the potential target genes of miR-21-5p. Six potential target genes were identified by overlapping differentially expressed genes (DEGs) from TARGET-OS dataset with predicted targets from StarBase database (Figure 5(a,b)). Among the 6 decreased genes, only ARHGAP24 was both significantly downregulated in MNNG/HOS and MG-63 cells than that in hFOB1.19 cells (Figure 5(c)). StarBase database was used to predict the binding sites between miR-21-5p and the 3’ UTR of ARHGAP24 mRNA (Figure 5(d)). Following that, luciferase reporter assay was performed to further confirm the direct relationship between miR-21-5p and ARHGAP24. The result suggested that miR-21-5p exerted an obvious suppressive impact on the luciferase activity in WT-ARHGAP24 constructs whereas no inhibitory impacts were found in mutation constructs (Figure 5(e)), suggesting that the direct binding relationship between miR-21-5p and the 3’UTR of ARHGAP24 mRNA. ARHGAP24 expression level was decreased in MNNG/HOS cells transfected with miR-21-5p mimic and increased in MG-63 cells transfected with miR21-5p inhibitor (Figure 5(f,g)). We concluded that miR-21-5p may exert its role by targeting ARHGAP24 directly.

(a) Overlapping differentially expressed genes (DEGs) from TARGET-OS dataset with predicted targets from StarBase database. (b) Volcano plot displayed DEGs in TARGET-OS dataset. (c) The target genes of miR-21-5p were examined by qRT-PCR in OS cell lines (MNNG/HOS and MG-63) and the hFOB1.19 cells. (d) MiR‐21-5p binding sequences in ARHGAP24 mRNA are displayed, and reporter gene plasmids were constructed. (e) The regulatory relationships were confirmed by luciferase reporter assay. (f) The expression of ARHGAP24 in transfected MNNG/HOS and MG-63 cells was examined by qRT-PCR. (g) The expression of ARHGAP24 in transfected MNNG/HOS and MG-63 cells was examined by western blot. *p < .05, **p < .01, ***p < .001

### Circ-LMO7 inhibited the proliferation and migration of osteosarcoma cells via regulating ARHGAP24 expression

**Figure 6.**
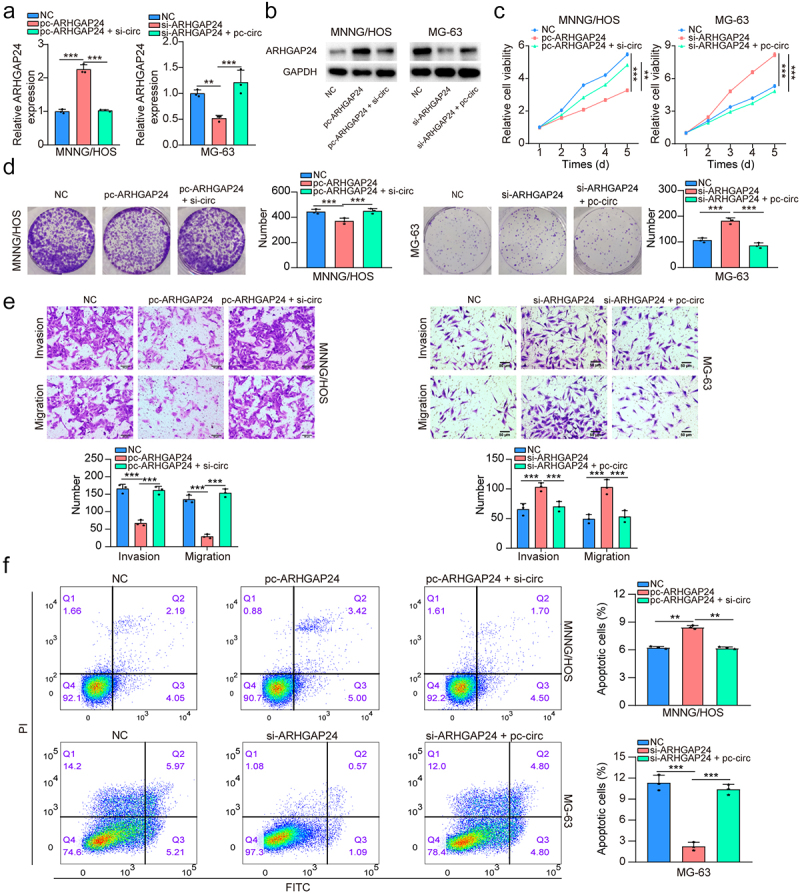
Circ-LMO7 inhibited cell proliferation and migration of OS via upregulating the expression of ARHGAP24.

To further verify the regulatory association between circ-LMO7 and ARHGAP24, we performed rescue experiments. pc-ARHGAP24 was transfected into MNNG/HOS cells and successfully up-regulated ARHGAP24, but circ-LMO7 silencing reversed the effect of pc-ARHGAP24 transfection (Figure 6(a,b)). ARHGAP24 expression was decreased in the ARHGAP24-knockdown MG-63 cells, but was partially reversed when cells were co-transfected with pc-circ-LMO7 (Figure 6(a,b)). Finally, followed by CCK-8, colony formation, transwell and flow cytometry assays, we demonstrated that ARHGAP24 overexpression was found to offset the impacts of circ-LMO7 down-regulation on OS cell growth, invasion, and migration, but promoted their apoptosis. ARHGAP24 downregulation partially reversed the inhibitory effects of circLMO7 overexpression on MG-63 cell growth, invasion, and migration, but suppressed apoptosis (Figure 6(c–f)). Collectively, these results concluded that circ-LMO7 inhibits proliferation, migration and invasion of OS cells via regulating ARHGAP24.

(a, b) The expression of ARHGAP24 was examined by qRT-PCR and western blot in ARHGAP24-overexpressing MNNG/HOS and MG-63 cells transfected with siRNAs for circ-LMO7 or not. (c, d) CCK-8 and colony formation assays were applied to assess the effect of circ-LMO7 on the proliferation of ARHGAP24-overexpressing MNNG/HOS and MG-63 cells; (e) Transwell assay was applied to assess the effect of circ-LMO7 on the invasion and migration of ARHGAP24-overexpressing MNNG/HOS and MG-63 cells. (f) Flow cytometry was applied to assess the effect of circ-LMO7 on the apoptosis rate in ARHGAP24-overexpressing MNNG/HOS and MG-63 cells. *p < .05, **p < .01, ***p < .001.

### Circ-LMO7 inhibited OS development through packaging into exosomes

**Figure 7.**
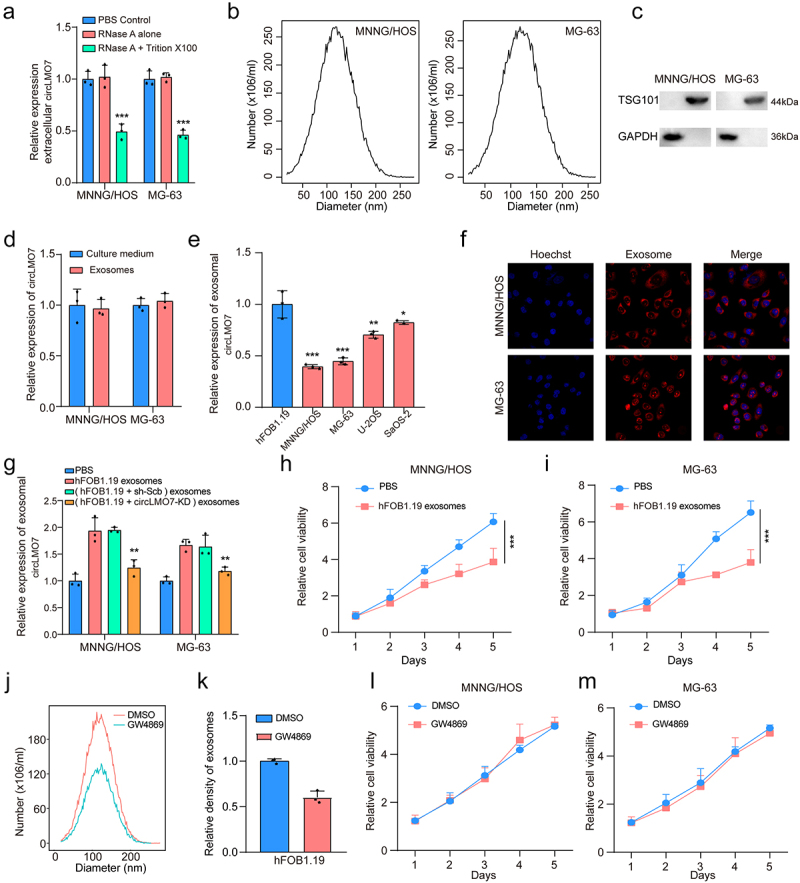
Circ-LMO7 inhibited OS development through packaging into exosomes.

We noticed that circRNAs could exert intercellular communication roles through the secretion of exosomes. We observed that extracellular circLMO7 expression was significantly depressed when MNNG/HOS and MG-63 cell lines were treated with RNase A and Triton X100, but unchanged when treated with RNase A lonely (Figure 7(a)). NTA analysis showed that the range of exosome diameter was 30‒200 nm (Figure 7(b)). We detected TSG101 protein expression to identify exosomes using western blotting (Figure 7(c)). These experiments confirmed that the exosomes were successfully obtained.

(a) Extracellular circ-LMO7 was degraded by treatment with RNAse A and Triton simultaneously; (b) Size distribution of exosomes were analyzed by nanoparticle tracking analysis (NTA); (c) Markers (TSG101) of exosomes were analyzed by western blotting (exosomes and exosome-depleted cell extracts); (d) Circ-LMO7 expression in culture medium and exosomes was analyzed by qRT-PCR; (e) Exosomal circ-LMO7 levels of hFOB1.19, MNNG/HOS, MG-63,U-2OS and SaOS-2 was analyzed by qRT-PCR; (f) Exosomes of hFOB1.1 was extracted and labeled with PKH26 dye followed by incubation with MNNG/HOS and MG-63 cells; (g) Exosomal circ-LMO7 levels of hFOB1.19 cells was decreased after treated with si-circ-LMO7; (h, i) Cell proliferation was determined by CCK-8 in MNNG/HOS and MG-63 cells incubated with exosomes from hFOB1.19 cells; (j, k) The size distributions and number of exosomes from hFOB1.19 cells treated with GW4869 were analyzed by NTA; (l, m) Cell proliferation was determined by CCK-8 in MNNG/HOS and MG-63 cells incubated with exosomes from GW4869-treated hFOB1.19 cells; *p < .05, **p < .01, ***p < .001.

Furthermore, we found the expression of exosomal circLMO7 levels was almost consistent to that in culture medium levels (Figure 7(d)), indicating that circ-LMO7 was mainly expressed in exosomes. Meanwhile, circ-LMO7 was down-expressed in OS cell lines compared to exosomes derived from hFOB1.19 cells (Figure 7(e)). We then extracted exosomes and labeled with PKH26 dye followed by incubation with MNNG/HOS and MG-63 cells. As shown in Figure 7(f), a strong red signal in MNNG/HOS and MG-63 cells indicated that the exosomes were taken up by receipt cells. Circ-LMO7 expression in exosomes derived from hFOB1.19 cells decreased significantly after transfected with si-circ-LMO7 (Figure 7(g)). After co-cultured with exosomes derived from hFOB1.19 cells, the cell viability of MNNG/HOS and MG-63 cells were significantly decreased (Figure 7(h,i)).

Next, we choose to investigate whether exosomes played a deterministic role, we blocked exosome production via using GW4869 (Figure 7(j,k)). Incubation with culture medium from hFOB1.19 cells treated with GW4869 failed to influence the cell viability of MNNG/HOS and MG-63 cells (Figure 7(l,m)). Based on above results, we supposed that extracellular circ-LMO7 inhibited OS cell viability through exosomes.

## Discussion

gIncreasing evidences demonstrated that circRNAs are critical contributors or suppressors to tumor proliferation and invasion, and could become molecular markers of various tumors.^19–21^ CircRNAs also have been reported to be involved in the progression of OS.^22^ For instance, Abnormal expression of CircECE1 acts as a contributor for OS via promoting cell proliferation and metastasis.^23^ A novel circRNA ROCK1 palys a suppressive role to retract proliferation and migration of OS cells.^24^ In this study, we discovered circ-LOM7 was packed in exosomes and down-regulated in OS cells, and evaluated the effect of circ-LOM7 on the proliferation and migration of OS cells.

Concerning the mechanisms of effects for circRNAs, one mode reported was the microRNAs (miRNAs) “sponges” activity of some exonic circRNAs. Studies have revealed that the mechanism of circRNAs to perform their function is binding with miRNAs to influence the expression of downstream target genes. The results of Yang’s study also showed that Circ_001422 is overexpressed in OS cell lines and promotes tumor development by modulating the miR-195-5p/FGF2/PI3K/Akt pathway.^25^ Higher circ_001422 expression accelerates proliferative and invasive potentials of OS through absorbing miRNA-195-5p.^25^ Our study found the biological function of circ-LOM7 in OS depended on absorbing miR-21-5p. Through bioinformatics prediction, ARHGAP24 was found to be a potential target gene of miR-21-5p. ARHGAP24 expression was positively regulated by circ-LOM7, but negatively regulated by miR-21-5p.

ARHGAP24 is a member of Rho GTPase-activating proteins which are composed of 748 amino acids involving cell processes. In addition, the interaction of miRNA and ARHGAP24 was studied before. For example, miR-590-5p modulates RCC cell line viability, apoptosis, migration, and invasion through targeting ARHGAP24.^26^ ALHGAP24 level pertains to cell processes, kidney metastasis as well as patients’ survival.^27^ Our study found that ARHGAP24 was highly expressed in OS tissues and cells. Overexpression of ARHGAP24 promoted invasive and migratory rates of OS cells. More importantly, ARHGAP24 overexpression reversed the promoted invasion and migration due to miR-21-5p knockdown, further demonstrating that ARHGAP24 was an important target gene for circ-LOM7 and miR-21-5p.

Considering that circRNAs could participate in the occurrence and progression of tumors through the secretion of exosomes,^28–32^ we also determined whether extracellular circ-LMO7 exerts its roles through incorporating into exosomes. We found circ-LMO7 could be packed into exosomes, and in accordance with the intracellular expression, the exosomal circ-LMO7expression of hFOB1.19 cells was higher than that in OS cell lines. So we choose exosomes extracted from derived hFOB1.19 cells to conducted co-culture experiments for heighten exosomal circ-LMO7 expression levels. The proliferation ability of OS cells was suppressed after treated with exosomes originated from hFOB1.19 cells. These results demonstrated that circ-LMO7 could be transmitted between cells through exosomes to promote OS cell proliferation.

In conclusion, circ-LOM7 promoted the expression of ARHGAP24 by targeting miR-21-5p and regulated cell growth, migration and invasion of OS. However, further research is needed on the mechanisms by which circ-LOM7 participates in the development of OS. This study provides new insights underlying the effects and regulatory mechanisms of circRNAs in OS, and moreover, provides preclinical data supporting the potential application of circRNA in OS treatment medicine.

## Data Availability

The datasets used and/or analyzed during the current study are available from the corresponding author on reasonable request.
